# Amplicon-based analyses of single-nucleotide polymorphisms reveal the genetic structure of a forest insect baculovirus

**DOI:** 10.1093/ve/veaf061

**Published:** 2025-08-23

**Authors:** Christian Oehlmann, Jiangbin Fan, Michael J Rihlmann, Hannes Lemme, Jörg Müller, Birgit Ruoff, Jörg T Wennmann, Johannes A Jehle

**Affiliations:** Federal Research Centre for Cultivated Plants, Institute for Biological Control, Julius Kühn-Institute (JKI), Schwabenheimer Str. 101, 69221 Dossenheim, Germany; Federal Research Centre for Cultivated Plants, Institute for Biological Control, Julius Kühn-Institute (JKI), Schwabenheimer Str. 101, 69221 Dossenheim, Germany; Key Laboratory of National Forestry and Grassland Administration for Control of Forest Biological Disasters in Western China, College of Forestry, Northwest A&F University, Yangling, Shaanxi, 712100, China; University of Kaiserslautern-Landau (RPTU), Gottlieb-Daimler-Str. 48, 67663 Kaiserslautern, Germany; Department of Forest Protection, Bavarian State Institute of Forestry, Hans-Carl-von-Carlowitz-Platz 1, 85354 Freising, Germany; Chair of Conservation Biology and Forest Ecology, Biocenter University of Würzburg, Glashüttenstraße 5, 96181 Rauhenebrach, Germany; Nationalpark Bavarian Forest, Grafenau, Germany; Federal Research Centre for Cultivated Plants, Institute for Biological Control, Julius Kühn-Institute (JKI), Schwabenheimer Str. 101, 69221 Dossenheim, Germany; Federal Research Centre for Cultivated Plants, Institute for Biological Control, Julius Kühn-Institute (JKI), Schwabenheimer Str. 101, 69221 Dossenheim, Germany; Federal Research Centre for Cultivated Plants, Institute for Biological Control, Julius Kühn-Institute (JKI), Schwabenheimer Str. 101, 69221 Dossenheim, Germany

**Keywords:** nucleopolyhedrovirus, insects, population genetics, modelling; biological control

## Abstract

Amplicon-based next-generation sequencing (aNGS) is a powerful tool in diagnostics and genetic studies. We developed an aNGS approach to study the population structure of the Lymantria dispar multiple nucleopolyhedrovirus (LdMNPV), a specific pathogen of the spongy moth *Lymantria dispar*, a devastating lepidopteran pest in European, Asian, and American deciduous forests. Naturally occurring pathogens, such as LdMNPV, are frequently reported to cause epizootics and a rapid decline of insect pest populations. DNA samples of pooled LdMNPV-infected larvae from forest regions in Northern Bavaria (Germany) were subjected to whole genome sequencing (WGS) and aNGS optimization. Then, five marker regions were identified in the genome of LdMNPV for PCR amplification, covering 21 highly specific single-nucleotide polymorphism (SNP) positions that enabled comprehensive analysis at the intra- and intersample levels. These markers were used in aNGS analyses of 70 single larvae collected in 12 forest sites, followed by SNP-based hierarchical clustering on principal components (HCPC). This approach identified three LdMNPV population clusters consisting of homogenous (pure) and heterogeneous (mixed) LdMNPV samples. To explain the genetic variability within each sample, a model based on linear optimization was developed and validated by comparing the predictions from aNGS and WGS data. The analyses showed that LdMNPV from Bavarian forests carried genetic variants highly similar to those present in the commercial product Gypchek®, developed for biocontrol. The distribution of genetic characteristics showed some trends of geographic and temporal prevalence, which are indicative of short-distance and long-distance transmission. The aNGS approach offers a fast, cost-effective, and comprehensive insight into the natural population structure of LdMNPV.

## Introduction

Baculoviruses are insect-specific double stranded DNA (dsDNA) viruses with covalently closed genomes of 80–180 kbp, which infect larval stages of Lepidoptera, Hymenoptera, and Diptera (Harrison *et al*. 2018). They are often noticed as natural epizootics of insect populations, especially at high host density (Cory and Myers 2003). Baculovirus field isolates may consist of single to numerous different genotypes (Redman *et al*. 2010, Bernal *et al*. 2013, Aguirre *et al*. 2019, Fan *et al*. 2021) mirroring viral population dynamics and genetic drift within and between the host larvae and time (Kennedy and Dwyer 2018). Variation within the genotype composition can affect the virulence or other phenotypic traits of the virus (Cory *et al*. 2005, Cabodevilla *et al*. 2011, Aguirre *et al*. 2019). The detection of genotype composition in an isolate is crucial for understanding the genetic diversity and therefore the pathogenic activity of the viral isolate. Viral genotype compositions can be quantified *via* the presence of single-nucleotide polymorphisms (SNPs) compared to a shared reference genome. This approach has been successfully followed with whole genome sequencing (WGS) using next-generation sequencing (NGS) and bioinformatic analysis (Chateigner *et al*. 2015, Fan *et al*. 2020, Wennmann *et al*. 2020, Fan *et al*. 2021, Wennmann *et al*. 2024, Wennmann 2025). Nevertheless, the comparison among viral isolates consisting of mixed genotypes is still a methodological challenge (Wennmann *et al*. 2024) and approaches using hierarchical clustering on principal components (HCPC) based on genomic SNP quantity and frequency were taken to identify the similarity of baculovirus isolates, e.g. for Bombyx mori nucleopolyhedrovirus (BmNPV) and Cydia pomonella granulovirus (CpGV) isolates (Fan *et al*. 2021).

An ecologically and economically important baculovirus is the Lymantria dispar multiple nucleopolyhedrovirus (LdMNPV), which causes natural epizootics in populations of the spongy moth *Lymantria dispar* L. and has been commercially registered as a biopesticide for spongy moth control in North American forests (Elkinton and Liebhold 1990, Podgwaite 1999). *L. dispar* (Insecta: Lepidoptera: Erebidae: Lymantriinae) is a widespread defoliating pest in the Northern hemisphere (Peterson *et al*. 2007, Paini *et al*. 2018). It has its evolutionary origin in Eurasia and was accidentally introduced to the USA in the 19th century (Picq *et al*. 2023). As an invasive pest in North America, *L. dispar* is known to exhibit two significant outbreak periodicities: a dominant cycle of 8–10 years and a less dominant period of 4–5 years (Haynes *et al*. 2009). During these outbreaks, the damage caused by larvae feeding on leaves of deciduous trees may result in complete defoliation of forests (Boukouvala *et al*. 2022). The total annual damage caused by *L. dispar* has been estimated at 3.2 billion US$ in North America alone (Bradshaw *et al*. 2016). In Central to South-Eastern Europe, the spongy moth exhibits outbreak periodicities ranging from 8 to 13 years (Hlásny *et al*. 2016, Lemme *et al*. 2025).

In 2018 and 2019, a mass outbreak of *L. dispar* occurred in Bavaria (Germany), which declined rapidly in 2020, supported by parasitoids and viruses (Leroy *et al*. 2023, Wolz *et al*. 2024). Since numerous larvae showed typical signs of infection with LdMNPV, such as swollen and liquefied larval bodies and hanging of larval cadavers from tree trunks and branches by their prolegs, a virus epizootic was hypothesized to be one of the major factors causing the collapse of the *L. dispar* populations. LdMNPV is assigned to the virus species *Alphabaculovirus lydisparis* (genus *Alphabaculovirus*, family *Baculoviridae*) (Harrison *et al*. 2018, van Oers *et al*. 2023). The genome of LdMNPV is ~157–164 kbp with a GC content of ~57.5% and encodes ~160 putative open reading frames (ORFs) (Kuzio *et al*. 1999, Krejmer-Rabalska *et al*. 2016). In addition, 13 homologous regions (*hrs*) were reported; *hrs* are highly repetitive regions, which may function as enhancers of gene expression and origins of replication. An exemplar isolate of *Alphabaculovirus lydisparis* is LdMNPV-5-6, which was obtained from a production lot of Gypchek®, a commercial LdMNPV formulation for spongy moth control (Slavicek *et al*. 1992). The US Environmental Protection Agency (EPA) registered Gypchek® in April 1978 for control of *L. dispar* in the USA. The LdMNPV isolate of Gypchek® is currently produced *in vivo*, consisting of a mixture of different LdMNPV genotypes. LdMNPV genotype mixtures can occur due to minor mutations within the genome (Harrison and Rowley 2015). Each transmission event between hosts and each replication cycle within hosts may alter the viral genotype composition through mutations, selection, and genetic drift. Effects such as transmission bottlenecks, modification of genotype frequencies by the host immune system, and demographic stochasticity can alter the composition of genotype mixtures (Kennedy and Dwyer 2018). Commonly used genomic regions to identify baculovirus isolates have been partial sequences of conserved genes, such as *late expression factor 8* (*lef-8*) or *late expression factor 9* (*lef-9*) (Jehle *et al*. 2006, Eberle *et al*. 2009). Harrison *et al*. (2014) attempted to distinguish between American, European, and Asian LdMNPV isolates based on a partial sequence of *lef-8* but failed to differentiate the European and the North American group, while the Asian LdMNPVs showed a higher diversity within the partial *lef-8* sequence. From these results, the authors hypothesized that the American *L. dispar* host and its NPVs were both derived from Europe, while the Asian LdMNPV isolates were more likely to originate from different subspecies of *L. dispar* (Pogue 2007). On the other hand, deWaard *et al*. (2010) showed that it is possible to distinguish between European and American *L. dispar* lineages as hosts on the basis of several molecular markers. This observation raised the question of whether differences exist between American and European LdMNPV isolates or whether the partial sequence of *lef-8* was simply insufficient to distinguish between those.

Here, we addressed the question of whether it is possible to establish markers for distinguishing naturally occurring baculoviruses at a local geographic scale. We developed a method to differentiate LdMNPV field samples from individual *L. dispar* larvae by identifying a minimum number of informative SNP positions that reflect the structure of local LdMNPV populations. The samples were characterized by amplicon-based NGS (aNGS) followed by HCPC. A linear optimization of the SNP distribution allowed us to decipher and quantify the prevalent LdMNPV subtypes in 70 individual larval samples.

## Materials and methods

### Lymantria dispar multiple nucleopolyhedrovirus and *L. dispar* samples

The commercial LdMNPV product Gypchek® (LdMNPV-Gyp) (production 2017) was obtained from Andermatt Biocontrol Suisse (Switzerland) in 2019.

**Figure 1:**
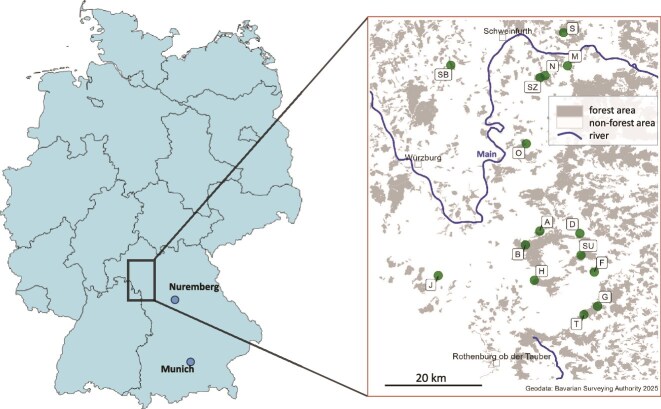
*Lymantria dispar* larvae were collected in the area of North Bavaria, Germany (left panel). Indicated are the cities of Würzburg, Schweinfurth, and Rothenburg ob der Tauber, the River Main, and the 15 collection sites A to SZ (green dots) (right panel).

Caterpillars of *L. dispar* were collected from North-West of Bavaria, Germany, from forests close to the cities Schweinfurt and Würzburg between April to May of 2019 and 2020 (Supplement Table S1). Living larval samples were collected from 15 sampling sides (A, B, D, F, G, H, J, M, N, O, S, and T, as well as SB, SU, and SZ) of high and low density of larval abundance across a transect of ~40 × 60 km (Leroy *et al*. 2021) (Fig. 1). In total, 488 larval stages [from first instar to fifth instar (males) or sixth instar (females) (L1–L5/L6)] were sampled, measured in body size, and stored individually in 96% Ethanol (EtOH) in 1.5 or 2 ml centrifuge tubes at −20 °C until further use.

### LdMNPV whole genome sequencing

WGS was performed on four LdMNPV samples: (i) LdMNPV-Gyp, (ii) LdMNPV-SB, (iii) LdMNPV-SU, and (iv) LdMNPV-SZ. The latter three samples were obtained from deceased and dried *L. dispar* larvae originating from three collection sites, i.e. Schraudenbach (SB), Sugenheim (SU), and Sulzheim (SZ). Dried larvae were hydrated in ddH_2_O overnight. Afterwards, the larvae were cut open and fat tissue was dissected using forceps and fluids containing viral occlusion bodies (OBs) were obtained using a Pasteur pipette. OBs were pelleted at 12 210 × g at 20 °C and washed using ddH_2_O three times. OBs were pooled in equal volumes from either three (SB and SZ) or two (SU) larvae originating from the same location. Pooling was performed to increase the likelihood of different variants in the samples.

For DNA extraction, the pelleted (12 210 × g, 20 °C, 10 min) OBs were dissolved by adding 300 μl of 0.1 M Na_2_CO_3_ and incubated at 37 °C for 30 min. The pH value was adjusted to pH 7 by adding 30 μl of 1 M HCl. RNA was digested by adding 1.4 μl RNase A (10 mg/ml) and incubation at 37 °C for 10 min. Then, 33 μl of 10% sodium dodecyl sulphate (SDS) and 4.5 μl proteinase K (20 mg/ml) were added. Samples were incubated at 37 °C for 1 h followed by a phenol/chloroform/isoamyl alcohol (25:24:1, v:v:v) extraction. Phenol residues were removed by an additional washing step using 300 μl chloroform. DNA was precipitated by adding 30 μl sodium acetate (3 M, pH 5.2) and 750 μl ethanol (96%) and incubation overnight at −20 °C. DNA was pelleted for 15 min, at 12 210 × g at 4 °C, and the pellet was washed using 70% EtOH. DNA was pelleted for 15 min, at 12 210 × g at 4 °C. The DNA pellet was dissolved in 50 μl ddH_2_O. About 100 ng DNA from LdMNPV-Gyp, -SB, -SU, and -SZ were sequenced (Illumina, paired-end, 150 nt) on a NextSeq 500 (StarSeq GmbH, Mainz, Germany) with about 5 M reads (~750 Mb) per sample.

### Determination of variable single-nucleotide polymorphism positions

Raw reads of LdMNPV-Gyp, -SB, -SU, and -SZ were processed using an in-house Galaxy server (Galaxy Version 20.01). Trim Galore v0.6.3 was applied for quality-filtering and adapter trimming (Krueger 2015). Only reads with an average quality Phred score (Q) > 30 and length > 50 nt were mapped against the reference LdMNPV-5-6 (GenBank accession no. AF081810) using BWA-MEM v0.8.0 (Li 2013) using default parameters. The reference LdMNPV-5-6 represents the consensus sequence of a plaque-purified isolate (Kuzio *et al*. 1999) and served as the common reference for interisolate SNP determination. From the four independent mappings, SNP positions were detected by using Samtools mpileup v2.1.1 (Li *et al*. 2009) with insertion/deletion calling disabled and a maximum per binary alignment map (BAM) depth of 12 000. All other quality filtering options and read group settings were kept at default. Nonvariant sites were removed from the analysis using BCFtools v1.0 (as part of Samtools, using a multiallelic and rare-variant caller with default parameters).

### DNA extraction for PCR survey of LdMNPV field samples

Total DNA was extracted from individual field-collected *L. dispar* larvae using Ron’s Tissue DNA Mini Kit (Bioron GmbH, Römerberg, Germany) with slight modifications for improved baculovirus genome isolation. Larvae were removed from the EtOH, in which they were stored, rinsed twice with H_2_O, and transferred individually to 1.5 ml centrifuge tubes. If the larvae had grown into a later larval instar (L4–L6), only a fraction of the larvae was extracted. Each sample was homogenized using a micro pistil in 200 μl lysis buffer (TCBL-1). Ten microliters of proteinase K (20 mg/ml) were added to the homogenate that was vortexed thoroughly and incubated at 55 °C for 90 min on a horizontal shaker (800 rpm). Then, 23 μl of 1 M Na_2_CO_3_ was added and further incubated for 30 min to lyse OBs possibly present in the sample. To adjust the pH to 7, 23 μl of 1 M HCl were added. DNA extraction was continued according to the manufacturer’s protocol, and DNA quantity and quality were measured using a NanoDrop 2000 (Thermo Fisher Scientific, USA) spectrophotometer.

### Oligonucleotide design

To identify genome-wide SNPs, the genome sequences of 18 LdMNPVs were aligned using Mauve Genomic Aligner Version 1.1.3 (Darling *et al*. 2004) in Geneious version 9 (Biomatters, New Zealand). These LdMNPV sequences originated from Spain (KT626570), South Korea (KF695050), Turkey (MF311096), the USA (AF081810, KU862282, KT626572), China (MT782113), Poland (KX618634), Japan (KT626571, MK089451), and Russia (KY249580, MK411293, KU862282, MK411291, MK411292, MN661137, KM386655, KP027546). A total of 15 candidate genes representing the conservative evolutionary characteristics of LdMNPV were chosen for designing oligonucleotide primer pairs for PCR, each spanning ~400 nt and covering between one and nine SNP positions (Table 1). All PCR oligonucleotide primers were designed using Geneious R9 using the sequence of an exemplar isolate of LdMNPV-5-6 (GenBank ID: AF081810) and could amplify ~400 bp fragment for each gene. Cytochrome C oxidase 1 (COX1) of *L. dispar* is used as a marker for spot-checking a successful DNA extraction.

**Table 1:** Oligonucleotide primers designed for the PCR amplification of regions with variable SNP positions. For each location, a forward (upper row) and reverse (lower row) was designed to cover variable SNP positions. The primer pairs #2, #6, #9, #11, and #12 were additionally supplied with Illumina standard sequencing adapters (forward: 5′-TCGTCGGCAGCGTCAGATGTGTATAAGAGACAG-3′, reverse: 5′-GTCTCGTGGGCTCGGAGATGTGTATAAGAGACAG-3′)

Primer pair (amplicon)	ORF	Gene name	Primer location on the reference genome^*^ (5′-3′)		Primer sequence (5′-3′)	GC (%)	Tm (°C)	Amplicon size (bp)	No. of SNPs on amplicon
#1	2	*pp78*	1615	1631	GCGCGGTCAGTTGGTAG	64.7	58.1	397	7
			2011	1994	CCGCTGCTGTTGGAGATC	61.1	58.2		
#2	5	*mucin-like protein*	7773	7791	GATCATCTGCACGACCGAG	57.9	58.1	404	9
			8176	8161	ACGTGGTGACCATTCT	50.0	51.7		
#3	22	*dna ligase*	21 793	21 810	CTCTACGACGCCGACGAC	66.7	60.0	395	2
			22 187	22 170	GCGTGATCCGAGACCACC	66.7	60.2		
#4	82	*desmo-plakin*	77 307	77 327	CTTCGTTCATTTCGACCTGCC	52.4	59.9	394	7
			77 700	77 680	GCCAAATTGTGTTCGGTCGAA	47.6	60.0		
#5	83	*dnapol*	78 504	78 521	GCATGACCTACGCCGACA	61.1	59.8	402	2
			78 905	78 888	ACTCGTGCACGTCCTTGG	61.1	60.0		
#6	88	*gp41*	84 030	84 051	GGAATATGTTCACCGACGAGGA	50.0	59.9	404	4
			84 433	84 412	CGCGCAAAAGTTTTACAACAAG	40.9	58.2		
#7	90	*tlp20*	85 430	85 448	TGTCCGCGTTCGAATCTCC	57.9	60.2	394	1
			85 823	85 805	GCGTACGTGACCCTTGACA	57.9	60.0		
#8	91	*vp91*	85 828	85 845	ATGTTGACCGTGTCGCTG	55.6	58.0	399	2
			86 226	86 210	CACATCGAGCCAGCCGT	64.7	59.8		
#9	92	*vp39*	89 570	89 590	TCGTCGATCATGATCTGCTCG	52.4	60.1	400	1
			89 969	89 950	TGAACCACAAAACCCTGGGC	55.0	61.0		
#10	109	*apsup*	107 935	107 953	AAAGCCACCATCACGTCGT	52.6	59.9	404	6
			108 338	108 320	CACGTTGGCGAACTTCTGC	57.9	60.1		
#11	130	*f protein*	126 332	126 350	GATCGAGGTGATCCCGCTG	63.2	60.0	403	2
			126 734	126 716	CACGGTGCCCACAAAGTTG	57.9	59.9		
#12	145	*sod*	139 272	139 289	TATGCGTCTTGTCCGGCG	61.1	60.2	404	5
			139 675	139 658	CGAGTTGCCGGTGGTCTT	61.1	60.0		
#13	156	*fgf*	152 300	152 320	AGTAGTAGCCGCTCATGTTGT	47.6	59.2	400	2
			152 699	152 679	GTAGAGGAGCTGAGCGAGAAC	57.1	59.9		
#14	160	*vef-2*	156 721	156 736	GCGCTCATCGACCAGC	68.8	58.1	40	4
			157 120	157 105	GCGCGAACGTCGTCTC	68.8	58.5		
#15	163	*hypo thetical protein*	160 324	160 339	TGCTGGTCAACGCGGA	62.5	58.7	393	4
			160 716	160 701	CGCACGCGTCCATCAC	68.8	58.8		

^*^Position on reference genome LdMNPV-5-6 (AF081810).

### PCR fragment amplification and purification

All PCRs were conducted following the standard protocol for GoTag® G2 Flexi DNA polymerase (Promega Corporation, Madison, USA) in either 25 or 50 μl total volume. After an initial step of denaturation (95 °C for 5 min), 35 cycles of denaturation (95 °C for 30 s), primer annealing (55 °C for 30 s), and elongation (72 °C for 1 min) were performed, followed by a final elongation step at 72 °C for 5 min. The successful amplification of PCR fragments was checked by gel electrophoresis (1% agarose, Tris-acetate-EDTA (TAE) buffer, 80 V for 1 h). Gels were stained with Midori Green Advanced (Nippon Genetics Europe, Düren, Germany). Amplified fragments were purified by following the manufacturer’s instructions for the Zymo PCR purification kit DNA Clean & Concentrator-25 (Zymo Research, CA, USA). The PCR fragments were eluted from the columns using ddH_2_O. The quality and quantity of the purified PCR fragments were determined using a NanoDrop 2000 (Thermo Fisher Scientific, USA) spectrophotometer.

### Nanopore sequencing of PCR fragments

The 15 purified PCR amplicons of samples LdMNPV-Gyp, -SB, -SU, and -SZ were prepared for sequencing in two different libraries. For each library, equimolar amounts of all 15 amplicons were pooled to obtain a total amount of 1 μg of DNA per sample. The DNA was prepared using the Ligation Sequencing Kit (SQK-LSK109) coupled with the Native Barcoding Expansion 1-12 (EXP-NBD104) (for sample LdMNPV-Gyp and -SB). In brief, 1 μg DNA per sample was end-prepped using the NEBNext Ultra II End-repair/dA-tailing kit (New England Biolabs, Ipswich, MA). Barcodes were ligated along with NEB Blunt/TA Ligase Master Mix (New England Biolabs, Ipswich, MA) for multiplexing. Equimolar amounts of each barcode sample were combined, followed by adapter ligation using NEBNext Quick T4 DNA Ligase and NEBNext Quick Ligation Reaction Buffer (New England Biolabs, Ipswich, MA). The final library was quantified using Quantus Fluorometer (Promega, Winsconsin, WI, USA). For all reactions, Eppendorf DNA Lobind tubes (Eppendorf GmbH, Hamburg, Germany) were used. Clean-up steps were conducted using Agencourt AMPure XP beads (Beckman Coulter, Indianapolis, IN, USA). Both libraries for LdMNPV-SU and -SZ, as well as the pooled library of LdMNPV-SB and Gypchek®, were loaded into FLO-MIN106D (R9.4.1) flow cells on an Mk1b device (Oxford Nanopore Technologies, Oxford, UK) and sequenced for 2 h, resulting in ~260k–440k reads per library. Basecalling was performed on an NVIDIA Jetson Xavier AGX developer kit (NVIDIA, Santa Clara, CA, USA) running on Ubuntu v19. Basecalling software was Guppy v4.4.0 implemented in MinKNOW v4.3.28. After basecalling, the reads were demultiplexed using qcat v1.1.0 (© 2018 Oxford Nanopore Technologies Ltd.) and quality-filtered using Nanofilt v2.7.1 (De Coster *et al*. 2018) with Q > 13. Reads were mapped against the reference genome of LdMNPV-5-6 (AF081810) with BWA-MEM v0.7.12 (Li 2013) using default parameters, resulting in a BAM file for each sequenced sample.

### Primer selection for single larvae sample analysis

After minimizing the number of required amplicons for sample characterization from 15 to 5 amplicons using an HCPC on sequencing data with the FactoMineR package v2.4 (Lê *et al*. 2008), the primer pairs #2, #6, #9, #11, and #12 (Table 1) were finally selected for PCR of single larval samples. The aim of the HCPC in this context was to group viral samples and compare the grouping results among different depths of information. If the groups stayed consistent between the original dataset (whole genome data) and the reduced dataset (15 amplicons, 5 amplicons), we considered the reduced dataset to be an adequate representation of the genetic variation of the whole genome. With this method, the dimensions of the datasets were reduced using a principal component analysis and the results were then assigned into clusters. The frequencies of the alternative nucleotide one were used for the HCPC as shown before (Fan *et al*. 2021). The alternative nucleotide 1 describes the most abundant nucleotide after the nucleotide present at this position in the reference genome. Frequencies of alternative nucleotides 2 and 3 were negligible, as previously shown in Wennmann *et al*. (2020). If not otherwise stated, the first three dimensions were considered for the analysis.

### Amplicon-based next-generation sequencing of field samples

The five amplicons of each sample were equimolarly pooled and sequenced (Illumina, paired-end, 250 nt) on a MiSeq reagent V2 (StarSeq GmbH, Mainz, Germany) with ~270k reads (~67 Mb) per sample. Afterwards, reads were trimmed for Illumina adapters and for a Phred quality score of 30 from each side. Reads shorter than 20 nucleotides were discarded.

The reads were mapped against the reference LdMNPV-5-6 (GenBank accession no. AF081810) using BWA-MEM v0.8.0 (Li *et al*. 2009, Li 2013). BAM files were processed in R using RSamtools v2.22.0 (Morgan *et al*. 2016). Nucleotide frequency of the 21 SNP positions was separately counted for each sample. The relative frequency of each nucleotide was then calculated by dividing the count of each nucleotide by the sequencing depth at that position. Plots were created using ggplot2 (Wickham 2011). Occurrence of different SNP patterns between Southern and Northern sampling sites was statistically analysed using Fisher’s exact test for each identified subtype.

### Hierarchical clustering on principal components

Agglomerative HCPC of the frequency of the alternative nucleotide 1 was performed, using the R package FactoMineR v2.11 (Lê *et al*. 2008) and Factoextra v1.0.7 (Kassambara and Mundt 2021). For identification of clusters, Ward’s method was considered and consequently taken for all analysis, due to the comparability of the clustering. *K*-means consolidation was performed with *K* resulting from the number of clusters determined in the hierarchical clustering by Ward’s method. The algorithm was iterated 10 times to find the appropriate shape of the cluster.

### Linear modelling of subtype prevalence

To prove the hypothesis that the majority of the samples were compositions of one or more pure viral subtypes, a model of the composition structure of single LdMNPV samples was developed as a linear programme (Karloff 1991), implemented in Python and solved with (Gurobi Optimization 2025).

## Results

### Selection of informative single-nucleotide polymorphisms

**Figure 2:**
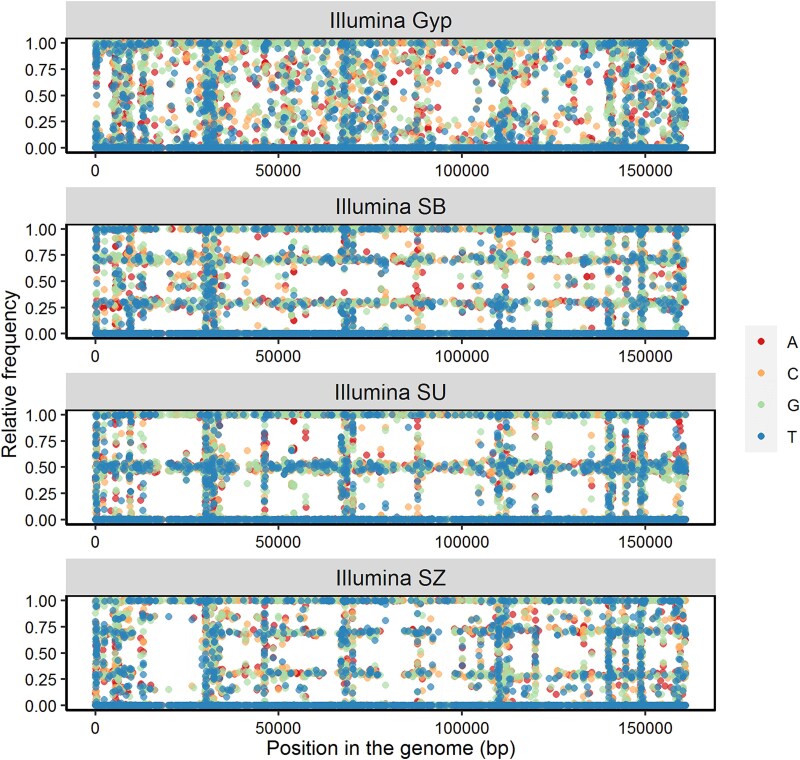
Relative nucleotide frequency at 2648 single-nucleotide polymorphism (SNP) positions of LdMNPV-Gyp (Gyp) and three local samples LdMNPV-SB, -SU, -SZ from Schraudenbach (SB), Sugenheim (SU), and Sulzheim (SZ), all sequenced with Illumina. Nucleotides are displayed as given in the legend (A = adenine, C = cytosine, G = guanine, T = thymine). Note: The vertical concentrations of SNPs over different positions in the genomes are typical for *homologous repeat* (*hr*) regions. Horizontal arrangements of SNPs indicate quantitative distribution of dominant genotype variants in LdMNPV-SB and -SZ (70:30) and in -SU (50:50).

The DNAs of the samples LdMNPV-Gyp, -SB, -SU, and -SZ were sequenced using Illumina sequencing (Table S2). Reads were aligned to the reference LdMNPV-5-6 and the distribution of nucleotides at each variable position was plotted (Fig. 2). In total, 2648 variable positions were identified. SNPs were scattered over the plots, although some were concentrated in a vertical (all samples) or horizontal arrangement (SB, SU, SZ). The SNP arrangements in vertical columns typically indicate the position of *homologous repeat (hr)* regions, as their sequences are more variable and because the sequence redundancy of *hr* complicates the read mapping in these regions. From the highly diverse SNP distribution pattern of LdMNPV-Gyp, it is hypothesized that it consists of a large number of different genotypic variants, whereas the horizontally even SNP distributions in LdMNPV-SB, -SU, and -SZ hint at a 70:30 mixture (in -SB and -SZ) and a 50:50 mixture (SU) of two predominant variants (Fig. 2).

**Figure 3:**
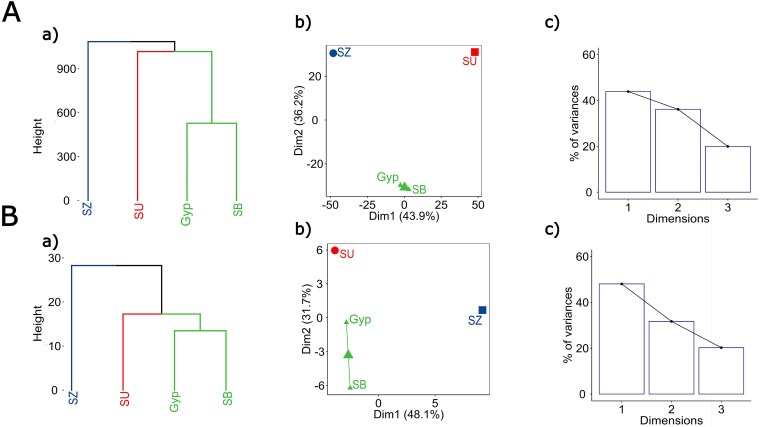
Hierarchical clustering on principal components (HCPC) of (A) the Illumina WGS data set (2648 SNPs) and (B) the 15-amplicon data set of LdMNPV-Gyp, -SB, -SZ, and -SU. (a) HCPC with Ward’s method revealed three clusters. (b) Classification of the genome data into three clusters was supported by a *K*-means consolidation. (c) The bar chart shows the percentage of variances explained by each dimension.

To reduce the complexity of the SNP distribution patterns and to cluster the samples by their similarity, an HCPC analysis was performed (Fig. 3A). The four samples clustered in three distinct groups. One cluster contained LdMNPV-Gyp and -SB, whereas -SU and -SZ were each placed in their own cluster. The dimensions Dim1 and Dim2 described 43.9% and 36.2% of the variance, respectively. The method of HCPC gives a valuable and robust insight into the similarity of viral isolates. If it is possible to reduce the amount of information needed to create the same similarity patterns in the HCPC, it would be more cost and computationally effective.

To prove if the similarity of the genomic SNP data of LdMNPV-Gyp, -SB, -SU, and -SZ could be reflected by a reduced number of SNPs in PCR amplicons, 15 genome regions were identified, which covered only 58 SNP positions within coding regions but excluded the variable *hr* regions (Table 1). PCR amplicons of the 15 regions were generated for the four samples and were Nanopore-sequenced (for details, see Table S3). After read processing and mapping of the 15 PCR amplicons to the LdMNPV-5-6 reference, again, three distinct clusters exhibiting the same pattern as the Illumina WGS data were identified by HCPC (Fig. 3B). The dimensions Dim1 and Dim2 accounted for 48.1% and 31.7% of the variance, respectively. These findings indicated that reducing the number of variants to 58 SNP positions could still robustly represent the similarity between the samples as covered by the original 2648 variable WGS positions.

### Determining the minimum amplicon number required for reliable clustering

In the next step, we aimed to determine a minimum number of variants that still represented the similarity between samples compared to the WGS data or the 15-amplicon sequencing data. To further reduce the number of SNPs needed to represent the similarity among the samples, all possible combinations between SNPs found on each of the 15 amplified fragments for maintaining the shape of the reference cluster (2648 SNPs) were tested. Since at least three SNPs had to be analysed together, all combinations using only one amplicon were discarded. The number of possible combinations of PCR fragments could be calculated by *n*-combination with *N* = 15, from the binomial coefficient:


$$ \left(\begin{array}{@{}c@{}}N\\{}n\end{array}\right) = \sum_{n=2}^{15}\left(\begin{array}{@{}c@{}}15\\{}n\end{array}\right)=32\ 752\ combinations $$

**Figure 4:**
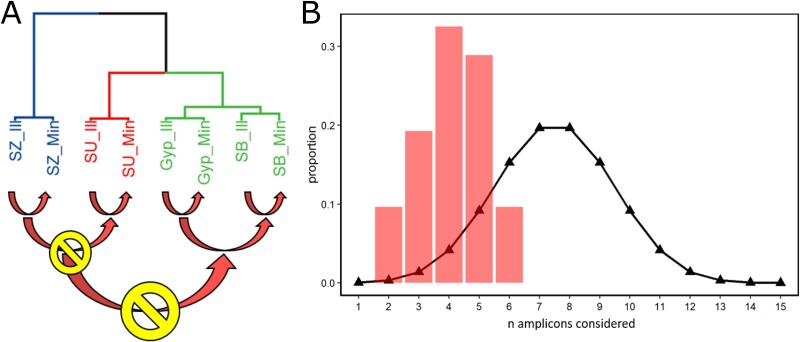
(A) Prerequisites for evaluation of all combinations of PCR amplicons. Combinations of amplicons generated from Illumina sequencing (ending with _Ill) and counterparts generated *via* Nanopore amplicon sequencing (ending with _Min) should always cluster together. Samples that were separate from each other before should not cluster together (e.g. SZ and SU, Fig. 3). (B) Triangles indicate theoretical distribution of combinations by n amplicons. Bars represent actual distribution of HCPC outcomes identical to the advanced reference cluster.

In addition, the Illumina and Nanopore amplicon sequencing data were analysed together to rule out systematic sequencing bias, as both methods were applied during the study. The SNP positions of each fragment were identified within the Illumina WGS. The Illumina and Nanopore amplicon data should therefore always be as close as possible within the same cluster. When this requirement is met, the sequencing technique is less likely to introduce systematic bias into the analysis. Therefore, the reference cluster was extended to the shape shown in Fig. 4A, still containing three main clusters.

If the HCPC algorithm was allowed to freely choose the number of clusters, a fourth cluster was introduced when more than six amplicons were considered at the same time (Fig. 4). As this was an unintended behaviour, only combinations consisting of two to six amplicons were found to be valid for the analysis. In total, 84 suitable amplicon combinations were identified. Based on this analysis, we considered the combinations of five amplicons as the most suitable ones, since this provided (together with four amplicons) the maximum number of possible combinations while covering a big-enough-to-be-stable number of SNPs. Only seven combinations based on five amplicons showed the required clustering matching of Fig. 3A (Table 2).

**Table 2:** Seven amplicon (primer pair) combinations able to represent the cluster shape of the information gained from SNPs of the whole genome of LdMNPV-Gyp, -SB, -SU, and -SZ. For primer pairs, see Table 1

Positive hit	c979	c1499	c1525	c1561	c1618	c1637	c1692
	#1	#2	#2	#2	#2	#2	#2
	#9	#5	#6	#6	#7	#7	#9
Primer pair	#11	#11	#7	#9	#9	#11	#11
	#12	#12	#11	#11	#11	#12	#12
	#15	#15	#12	#12	#13	#13	#13

From these combinations, all combinations containing primer pairs #13 were excluded because some unreliability within the PCR was experienced during the analyses. Also, the combinations c979, c1499, and c1525 (Table 2) were excluded because of the absence of either primer pair #2, #11, or #9. Due to their high abundance in the final combinations, it was clear that these primers were important for the correct clustering. Hence, primer combination c1561 with the primer pairs #2, #6, #9, #11, and #12 was selected for further analyses due to its consistent amplification and its ability to reflect sample similarity, as demonstrated in the HCPC utilizing SNPs of the Illumina WGS data set. The use of these five primer pairs resulted in amplicons covering 21 SNP positions.

### Analysis of field-collected viral samples

**Figure 5:**
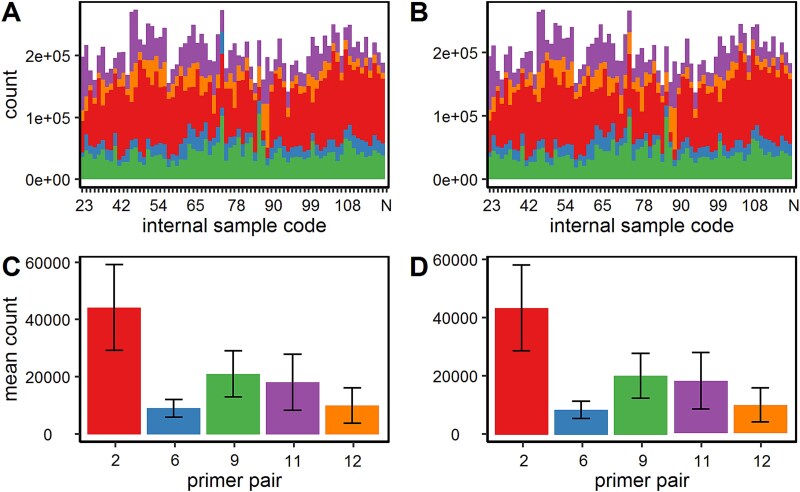
Read counts and read distribution of 70 + 4 amplicon-sequenced LdMNPV samples. Read counts for both forward and reverse reads per sample are given for (A) raw data and (B) trimmed data. The mean values and standard deviation (vertical bars) of reads for each PCR marker were calculated for (C) raw data and (D) trimmed data. Trimmed data refer to read data with sequencing adapters removed and trimmed ends for a Phred quality score > 30. Negative control of the sequencing was marked as N on the *x*-axis.

Out of the 488 field-collected larvae, 103 LdMNPV-positive samples (tested with primer pair #9) from 12 out of 15 sampling sites were identified, of which 70 samples (plus four samples LdMNPV-Gyp, -SB, -SU, and -SZ) resulted in PCR amplicons for all five primer pairs. Pooled fragments by amplicons of each sample were subjected to Illumina sequencing. The distribution of read counts for each primer pair and sample is given in Fig. 5. The read counts generated with primer pair #2 had the highest abundance, followed by the amplicons #9, #11, #12, and #6 (Fig. 5). After adapter trimming and quality filtering, the proportion of amplicon abundance stayed the same, indicating reliability of the sequencing itself. The abundance of amplicons was similar throughout all sequenced samples (Fig. 5). Potential PCR amplification bias between amplicons was ruled out by sufficient sequencing depth.

**Figure 6:**
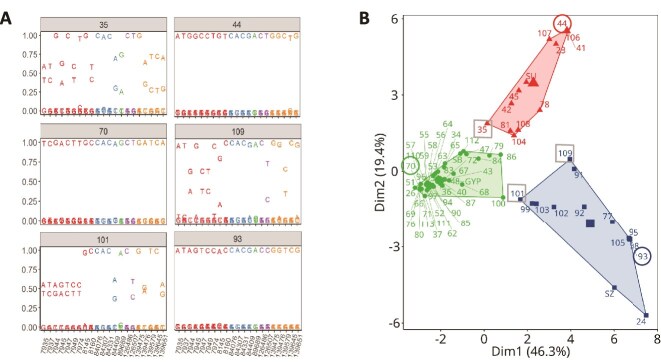
(A) Examples of SNP frequency plots of six samples with homogenous patterns (samples 70, 44, and 93) and heterogeneous patterns (samples 109, 35, and 101). Different nucleotide colours represent different amplicons, starting with #2 (red) and ending with #12 (yellow) (see Fig. 5). (B) Factor map resulting from hierarchical clustering on principal components (HCPC) of aNGS data from 70 field-collected LdMNPV samples and LdMNPV-Gyp, -SB, -SU, and -SZ. It resulted in three clusters, A (green), B (red), and C (blue). Edges of clusters A–C represent homogenous samples, e.g. sample 70 equals subtype A.1, sample 44 = B.1, and sample 93 = C.1, while the samples in between the outer samples are mixtures of them. The examples of pure and mixed samples from (a) are circled and squared, respectively, in (b).

Within the 74 aNGS-analysed LdMNPV samples, homogeneous and heterogeneous SNP patterns could be detected (Fig. 6A). In the case of samples with homogeneous SNP patterns (e.g. sample 44, 70, and 93 in Fig. 6A), it can be assumed that a single dominant genotype was present in the LdMNPV sample to produce this homogenous pattern (=subtype).

To assess the similarity between the different LdMNPV samples, an HCPC analysis was performed using the aNGS data set of the five amplicons of each sample (Fig. 6B). Three clusters, A, B, and C, comprising 47, 13, and 14 LdMNPV samples, respectively, were identified. Within the factor map, cluster A was the densest one, while cluster C appeared to be the least dense, indicating a higher similarity among the samples in cluster A than samples in cluster B or cluster C (Fig. 6B). Besides the typical harsh cut-off of a cluster, the dissimilarity between samples increased with distance, within and between clusters, in the factor map.

The aNGS data contained highly homogenous samples within the three clusters A–C, which differed from each other by the variation of one or more SNPs, resulting in the cluster defining types A, B, and C and related subtypes (Table 3). The main difference among the subtypes A.1–A.4 were single-nucleotide changes in three positions 84 076, 84 409, and 89 689, whereas the subtypes B.1–B.3 differed in up to seven positions from each other. Type C.1 showed the least similarity with the reference, with only seven identical SNP positions. In general, the subtypes A.1–A.4 seemed to be more uniform than the subtypes B.1–B.3.

**Table 3:** Nucleotide composition of the homogenous variants (=subtypes) found among the amplicon-sequenced samples. Colours indicate the different amplicons on which the SNP is located. Its positions in the LdMNPV-5-6 reference genome (AF081810) are given at the top of the columns. Horizontal rows show the three types A to C with their respective subtypes. Type names A–C refer to the HCPC clusters to which the subtypes were assigned (see Fig. 6). A, T, G, and C represent adenine, thymine, cytosine, and guanine residues at the given position, respectively. ^+^Position represents the consecutive number of the 21 SNPs. ^*^The bottom line ‘Error (%)’ indicates for each SNP position the percentage of the 74 analysed samples in which the maximum error in the linear model for the aNGS data was >10%

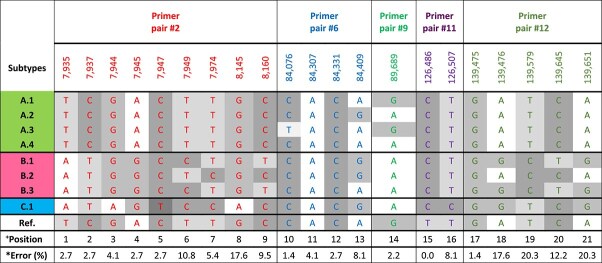

### Modelling the composition of mixed subtype samples

Based on the assumption that the mixed variants within the LdMNPV field samples consisted of two or more homogenous subtypes in the samples, it was hypothesized that the mixed samples could be explained as linear combinations of different pure subtypes. To approach this goal, a mathematical model based on linear optimization was developed to identify the subtypes within mixed populations. The model is based on describing the SNP composition of a given sample $I$ as a $n\times 4$ matrix with $n=21$ rows (number of SNP positions) and 4 columns containing the relative frequency of the nucleotide (A, C, G, T) at each position.


$$ I=\left(\begin{array}{@{}cccc@{}}{s}_{1A}& {s}_{1C}& {s}_{1G}& {s}_{1T}\\{}\vdots & \vdots & \vdots & \vdots \\{}{s}_{nA}& {s}_{nC}& {s}_{nG}& {s}_{nT}\end{array}\right)\in{\left[0,1\right]}^{n\times 4} $$

A homogenous (=pure) variant can be represented as a matrix containing only 0 and 1 values with exactly one entry equal to 1 in each row specifying the nucleotide at the corresponding position.

The matrix


$$ {V}^1=\left(\begin{array}{@{}cccc@{}}1& 0& 0& 0\\{}0& 0& 0& 1\\{}0& 0& 1& 0\\{}0& 0& 1& 0\\{}\dots & \dots & \dots & \dots \end{array}\right)\in{\left[0,1\right]}^{21\times 4} $$

is then a representation of the pure subtype ATGG… with $n=21$ SNP positions. Let ${V}^1,\dots, {V}^{{{n}}_{{pure}}}$ denote the representation matrices of the ${{n}}_{{pure}}=8$ identified pure subtypes. Each sample $I$ can then be described as a mixture of the pure subtypes using the formula


$$ \mathbf{I}=\sum_{\mathbf{i}=\mathbf{1}}^{{\mathbf{n}}_{{pure}}}{\mathbf{x}}_{\mathbf{i}}{\mathbf{V}}^{\mathbf{i}}+\mathbf{F} $$

with the continuous optimization variables ${x}_i\in \left[0,1\right]$ for $i=1,\dots, {n}_{{pure}}$, indicating the ratio ${x}_i$ of the pure subtype ${V}^i$ in the sample $I$. As a sample may also contain further unknown pure variants, an error matrix $F$ was introduced to the above formula. An estimation of the composition of the sample is computed such that the sum of all absolute entries of the error matrix is minimal:


$$ {min}\sum_{j=1}^n\sum_{X\in \left(A,C,G,T\right)}\left|{F}_{jX}\right| $$

The problem to compute the composition of each sample $I$ can be modelled as the following *linear programme* (*LP*):


$$ {\displaystyle \begin{array}{lll}{min}& \sum\limits_{j=1}^n\sum\limits_{X\in \left(A,C,G,T\right)}\left|{F}_{jX}\right|& \\[10pt] {}{subject}\ {to}& \sum\limits_{\mathbf{i}=\mathbf{1}}^{{\mathbf{n}}_{{pure}}}{\mathbf{x}}_{\mathbf{i}}{\mathbf{V}}_{\mathbf{jX}}^{\mathbf{i}}+{\mathbf{F}}_{\mathbf{jX}}={\mathbf{I}}_{\mathbf{jX}}& {for}\ {all}\ \left(j,X\right)\in \left(1,\dots, n\right)\times\\{}&&\quad \left(A,C,G,T\right),\\{}& 0\le{x}_i\le 1& {for}\ {all}\ i=1,\dots, {n}_{{pure}}.\end{array}} $$

A standard linearization approach can be applied to the objective function to eliminate the absolute values. As only continuous variables are considered, the problem can be solved efficiently with the simplex algorithm.

After the linear programme was implemented in Python and solved with Gurobi (Gurobi Optimization, 2025) for all analysed LdMNPV samples, for 40 out of the 70 single larvae LdMNPV samples (57%), the largest absolute entry (=maximum error) of the error matrix F was <0.1, and 92% of all data points (21 × 74 matrix) were estimated with a maximum error of <0.1 (Table S5). Some of the SNP positions (e.g. position 8 or 19–21), however, were modelled with a higher maximum error than others (Table 3, bottom line, ^*^Error) indicating that the prediction in these positions in the linear model contributed most to the observed error and that the total of samples must contain further variability, especially in these positions.

The maximum error was smallest for those isolates located towards the most extreme points of the clusters and increased when the samples had an intermediate position in the clusters. For example, sample 35 was predicted as a combination of 41% A.1, 7% B.1, 23% B.2, and 29% B.3, with a maximum error of 0.336, whereas sample 101 was predicted to comprise a mixture of 3% A.1, 41% A.4, and 55% C.1. The SNP distribution of sample 101, however, cannot be explained without a type C variant, which has a G residue instead of an A residue at position 8145, apparently leading to the high maximum error of 0.528 (see Fig. 6 and Table 3).

### Detectable subtypes in comparisons among sequencing technologies

**Figure 7:**
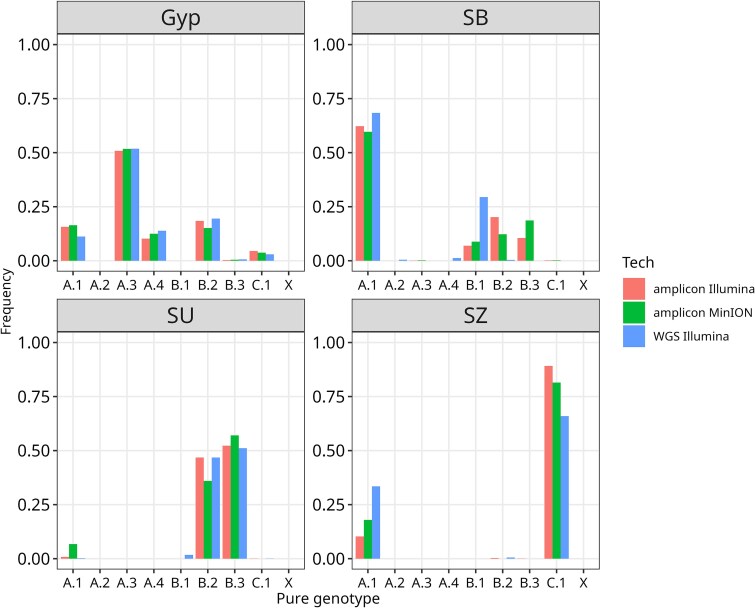
Modelling the presence of different subtypes found in LdMNPV-Gyp and in field samples -SB, -SZ, and -SZ, derived from Illumina and Nanopore amplicon sequencing (aNGS) and Illumina whole genome sequencing (WGS).

Since the initial field samples (LdMNPV-SB, -SZ, -SU) were collected in the same area as the identified homogenous subtypes, and since Gypchek® itself showed a high similarity to the majority of samples of cluster A (Table 3), we further compared the predicted prevalence of homogenous subtypes detectable in LdMNPV-Gyp, -SB, -SU, and -SZ using the datasets from Illumina aNGS, Nanopore aNGS, and Illumina WGS (Fig. 7). As exemplified for LdMNPV-Gyp (Fig. S2), the SNP distributions obtained from the three methods were highly similar. Within LdMNPV-Gyp, there are five SNP subtypes, A.1, A.3, A.4, B.2, and C.1, explaining >99% of the observed distribution and a maximum error of 0.276 (Fig. 7, Table 4, Table S5). LdMNPV-SZ was predicted to comprise a mixture of A.1 (33.4%) and C.1 (65.8%). LdMNPV-SU mainly consisted of B.2 and B.3 of ~50:50 for all three methods, whereas SU was predicted to be a mixture of A.1 and C.1. Though with some variations, the ratios of subtypes explaining the mixture were highly consistent across the different sequencing methods, which demonstrated the reliability of the experimental approach. For the LdMNPV-SB, the picture was less clear. Whereas A.1 was predicted with a ratio of 0.66, the WGS and aNGS varied for predicting the other subtypes B.1, B.2, and B.3 (Fig. 7). Sample LdMNPV-SB was classified in cluster A but was positioned relatively close to cluster B (Table 3). Therefore, the mixture of A and B subtypes is reasonable but less stably resolved than in the other three samples. The comparison of sequencing methods given in Fig. 7 further showed that in most cases, Illumina amplicon and Illumina WGS rendered similar subtype attributions, whereas Nanopore sequencing added some variability, suggesting that the sequencing method added only a small bias to the analyses and that the Illumina amplicon sequencing generated highly accurate nucleotide distributions when compared with the original genomes.

**Table 4:** Modelled composition of LdMNPV samples based on Illumina aNGS. The estimated proportions (%) of subtypes A.1–C.1. for the pooled samples Gyp, SB, SU, and SZ (amplicon Illumina) are indicated. For the single larval samples from different collection sites (compare Fig. 1), the estimated rates were classified into >95% (upper bold text), 10%–95% (middle italic text), and 5%–10% (lower normal text). Numbers in brackets indicate the number of samples per collection site. If the subtype was not found within the isolate a hyphen is shown.

		Associated subtypes							
		A.1	A.2	A.3	A.4	B.1	B.2	B.3	C.1
Product/Pooled larvae	Gyp	15.8%	–	50.8%	10.1%	–	18.5%	–	4.5%
	SB	62.2%	–	–	–	6.9%	20.2%	10.5%	–
	SU	–	–	–	–	–	44.8%	54.1%	–
	SZ	10.2%	–	–	–	–	–	–	89.2%
Single larval samples (Northern collection sites)		–	**2**	–	–	–	–	–	–
	S (9)	*5*	*–*	*1*	*–*	*1*	*1*	*1*	*–*
		2	2	–	–	–	–	–	3
		**2**	–	–	–	**2**	–	–	**3**
	N (19)	*6*	*1*	*–*	*1*	*3*	*4*	*3*	*5*
		3	–	1	–	1	2	–	–
		–	–	–	–	–	**1**	–	**1**
	SB (2)	–	–	–	–	–	–	–	–
		–	–	–	–	–	–	–	–
		–	–		–	–	–	–	–
	SZ (4)	*2*	*–*	*–*	*–*	*–*	*3*	*2*	*1*
		–	–	1	–	–	–	–	–
		**5**	–	–	–	–	–	–	–
	O (14)	*9*	*1*	*–*	*–*	*1*	*3*	*3*	*–*
		–	–	–	1	3	1	3	2
Single larval samples (Southern collection sites)		–	–	–	**2**	–	–	–	–
	B (2)	–	–	–	–	–	–	–	–
		–	–	–	–	–	–	–	–
		–	–	–	**5**	–	–	–	–
	D (6)	–	–	–	*1*	–	–	–	–
		–	–	–	–	–	–	–	–
		–	–	–	–	**1**	–	**1**	–
	SU (3)	–	–	–	*–*	*–*	*1*	*–*	–
		–	–	1	–	1	–	–	–
		–	–	–	–	–	–	–	–
	F (5)	*–*	*–*	*–*	*5*	*–*	*2*	*–*	*5*
		–	–	–	–	–	1	–	–
		–	–	–	–	–	–	–	–
	H (1)	*–*	*–*	*–*	*–*	*–*	*–*	*–*	*1*
		1	–	–	–	–	–	–	–
		–	–	–	–	–	–	–	–
	G (1)	*–*	*–*	*–*	*–*	*1*	*–*	*–*	*–*
		1	–	–	–	–	–	–	–
		–	–	**1**	–	–	–	–	–
	J (3)	*2*	*–*	*–*	*–*	*1*	*–*	*–*	*1*
		–	–	–	–	–	1	2	1

### Distribution of LdMNPV subtypes in the field

The analysis of the 70 single larva samples revealed that 26 larvae (37% of samples) contained more or less pure subtypes (>95% of a single subtype in a sample), of which A.1 (10%) and A.4 (10%) were the most frequent and B.2 and B.3 (1.4%) were the least abundant ones (Table 4). Pure A.1 appeared only in the northern sampling blocks, whereas A.4 dominated the southern ones (here only in the blocks B and D). Pure C.1 subtypes occurred only in the north (blocks SB and N). All larvae from block F (south) were infected by a mixture of A.4 and C.1; some of these samples also contained B.2. Also, block O (north) was highly consistent, with A.1 present in all samples and about two-thirds of the samples comprising mixtures of A.1 with other A, B, and C subtypes. Notably, the collections of 2019 and 2020 in block O comprised highly similar subtype compositions suggesting a local stability of subtypes over the sampling years (Table S5). With the exception of two samples (72, 90), the subtype A.4 mainly occurred in the southern blocks. The observed uneven geographic distribution of both A.1 and A.4 is statistically highly significant (*P* < .0001, two-tailed Fisher’s exact test).This distribution of subtypes in single larvae is also well represented in the pooled samples LdMNPV-SB, -SU, and -SZ, where for the northern samples -SB and -SZ also A.1, B.1, and C.1 was predicted, whereas -SU was explained by a combination of B.2 and B.3.

## Discussion

Classical methods to analyse the genetic diversity and population genetics of large DNA viruses, such as baculoviruses, in field isolates include comparative restriction analyses, use of PCR markers, and more recently WGS of viral DNA (Rezapanah *et al*. 2008, Erlandson 2009, Chateigner *et al*. 2015, Fan *et al*. 2021, Zanella-Saenz *et al*. 2022). These methods, however, have their limitations in the lack of quantifiable characters or they are cost and computationally intensive and less suitable for studying large numbers of samples. In contrast, marker-assisted detection of variability in virus samples provides a new opportunity in analysing virus population structure and population dynamics. Such methods were developed for some clinically important virus groups, e.g. SARS-Cov-2, orthohantavirus, and influenza virus (Taylor *et al*. 2020, Nasereddin *et al*. 2022, Croville *et al*. 2024) but rarely for large DNA viruses. Here, we provide a straightforward method for applying SNP markers in LdMNPV, which reflects its genetic diversity. Starting from WGS data of LdMNPV-Gyp and three pooled LdMNPV samples from Bavaria, we selected five PCR marker regions (#2, #6, #9, #11, #12) that were considered as suitable to represent the local population structure of LdMNPV (Figs 2 and 3, Table 2).

Primer pair #2 targets Ld-ORF 5, which seems to be specific to LdMNPV (Kuzio *et al*. 1999). Ld-ORF5 encodes a mucin-like protein and has been identified for all LdMNPV genomes sequenced so far (Harrison *et al*. 2014). This ORF was also identified in Lymantria xylina nucleopolyhedrovirus (Nai *et al*. 2010). It was not found in any other baculovirus genome (Rohrmann 2019), and its function was not further characterized. Primer pair #6 is specific for Ld-ORF 88, which encodes the protein GP41. As a structural protein, it serves as an O-glycosylated tegument protein. It is located between the nucleocapsid and the virion envelope (Whitford and Faulkner 1992). Furthermore, it was found to be associated with ODVs of different NPVs (Pan *et al*. 2005, Wang *et al*. 2010) and for other NPVs associated with BVs and ODVs (Hou *et al*. 2013). GP41 is essential for BV and ODV assembly (Li *et al*. 2018). Primer pair #9 aligns to a sequence within Ld-ORF 92, encoding the major capsid protein VP39. As one of the 38 baculovirus core genes, it is present in all baculovirus genomes sequenced to date (Rohrmann 2019). It is one of the most abundant proteins associated with BV production of AcMNPV (Wang *et al*. 2010), and functional studies revealed that it is required for DNA packaging and the assembly of nucleocapsids (Katsuma and Kokusho 2017). Primer pair #11 targets a sequence within the *f protein* gene located in Ld-ORF 130. This gene occurs in the genome of nearly all baculoviruses, except gammabaculoviruses (Rohrmann 2019). Together with ORF 8 of Spodoptera exigua nucleopolyhedrovirus, the LdMNPV ORF 130 was identified as the first functional analogues of GP64, the fusion protein employed by AcMNPV and other group I alphabaculoviruses (IJkel *et al*. 2000, Pearson *et al*. 2000). Primer pair #12 targets the Ld-ORF 145, which encodes a homologue of the superoxide dismutase (SOD). It appears to be nonessential for AcMNPV but might facilitate infection of hemocytes (Rohrmann 2019).

By using these five oligonucleotide primer pairs, PCR amplicons, and aNGS data from 70 individual *L. dispar* larval samples were generated. The sequencing depth of 5000–10 000-fold coverage at any given SNP position in each sample allowed an in-depth analysis of the SNP distribution. When applying HCPC analysis to the SNP distribution data, the samples grouped into three clusters A–C representing the genetic variation among the samples (Fig. 6). The more similar the structure of nucleotide frequency within the SNP frequency plots was, the narrower the samples were placed within the factor map. The first two HCPC dimensions could explain about two-thirds of the variance, which results in an appropriate conservation of information. Two-thirds of the samples grouped within cluster A indicating a low diversity among these specimens and a high prevalence of this type compared to the types B and C.

Apparently, LdMNPV-Gyp, which originated from North America, was placed right in the centre of cluster A, indicating a high similarity between LdMNPV-Gyp and the neighbouring samples of cluster A. Another question arising from this study is whether it allows the determination of geographic footprints and identification of certain isolates in the field. An attempt to distinguish geographic isolates of LdMNPV from different geographical origins rendered some ambiguous results (Harrison *et al*. 2014). The target genes used in their PCR analyses belong to the most highly conserved baculovirus genes, such as *polh*, *lef-8,* and *lef-9,* and may not be fully suitable for isolate differentiation within a given baculovirus species. Recent WGS-based analyses proposed a narrow relationship of North American and European LdMNPV isolates (Harrison *et al*. 2016, Gencer *et al*. 2023). This hypothesis is supported by finding LdMNPV types similar to the North American LdMNPV-Gyp in the Bavarian samples. Our approach further allowed the resolvation of sample differences even over a very limited area and should also be able to differentiate between samples separated by large distances.

The SNP distribution data of the different samples revealed homogeneous and heterogeneous SNP frequency patterns with a total of eight homogenous subtypes, which likely represent samples with a single highly dominant genotype (Table 3). Genotype mixtures are typical for naturally occurring baculovirus isolates, especially alphabaculoviruses, which co-occlude a large number of virions in a single OB and in which virions may contain several nucleocapsids, as is the case with multiple nucleopolyhedroviruses, such as LdMNPV (Slavicek 1991, Redman *et al*. 2010, Podgwaite *et al*. 2013, Beperet *et al*. 2021). Each LdMNPV sample with unevenly distributed SNP patterns must be a mixture of two or more genotypes, whereas samples with homogenous SNP patterns were considered as pure genotypes, though variation might be present in genome regions not represented in the amplicons. On the other hand, it is notable that 37% of samples carried a highly dominant single subtype, proposing that pure genotype infections are rather common under natural infection conditions.

Some of the samples’ mixed SNP patterns could be identified visually as a mixture of two subtypes. This observation led us to assume that the mixed subtype samples might be explained by a combination of pure subtypes found in the area. To prove this hypothesis, the composition of each sample was modelled as a linear combination of these subtypes A.1–C.1 by minimizing the error matrix in an LP. With these underlying assumptions, this model could explain the composition of nearly 60% of single larvae samples with a maximum error smaller than 10%. Of course, the more complex the SNP distribution was, the larger the maximum error of the prediction became, indicating that further factors contributed to the observed genetic diversity in the samples. Such factors could be the presence of further subtypes not identified in our samples and recombination between subtypes, which were not covered by the LP. Single positions with high maximum error are indicative of positions not following the model.

Additional heterogeneity within virus genomes might derive from highly variable genome regions, e.g. *hrs* and *bro* genes in baculoviruses, which are known as hotspots of genomic variability (Chen *et al*. 2001, Zhang *et al*. 2005). These highly variable regions may not be sufficiently stable to be an informative representation of the viral genome population structure and were therefore not included when target sequences for PCR amplification were selected. A possible direction of future research would be to develop algorithms based on integer linear programming to determine not only the composition of the samples but also further subtypes themselves.

The LP-based modelling of the observed SNP patterns predicted the subtype composition of the majority of samples with remarkable accuracy. For instance, the isolate LdMNPV-Gyp was modelled as a combination of at least five subtypes: A.1, A.3, A.4, B.2, and C.1 (Fig. 7, Table S5), which correlates well with the fact that LdMNPV-Gyp is a highly complex mixture of genotypes as demonstrated by WGS (Fig. 2) and as reported previously (Slavicek *et al*. 1995). The consistency of the amplicon sequencing approach was further proven when its results were compared with the outcome of the WGS with over 2648 SNP positions (Fig. 2). Although LdMNPV-Gyp is a mixture of numerous genotypes, the composition of samples -SZ and -SU was reasonably represented by different sequencing methods (Fig. 7). Only the prediction of sample SB seemed to be less robust, as Illumina WGS proposed a ~70:30 mixture consisting of A.1 and B.1, whereas Illumina and Nanopore aNGS required additional subtype(s) to explain the amplicon SNP patterns adequately (Figs 2 and 7, Table 4). The comparison of the Illumina WGS and Illumina amplicon sequencing methods further demonstrated the methodical reliability as the SNP distribution and the subtype predictions were highly consistent (Fig. 7). From this finding, it can be further concluded that the PCR used to generate the amplicons added minimal bias to the analysis and that the population structure of the viruses was well represented by the SNP distributions in the amplicons.

The aNGS approach allowed the identification of LdMNPV samples originating from single spongy moth larvae collected in the field without the necessity to further propagate the virus *in vitro* or *in vivo* (Eberle *et al*. 2012). Such propagations in the laboratory may significantly affect the genotype composition as any change of the genetic and cellular background of the propagating environment might result in mutation and selection of viral variants which are not identical to the original inoculum (Pijlman *et al*. 2001, Simón *et al*. 2004, Cory *et al*. 2005, Erlandson 2009, Larem *et al*. 2019).

The genotypic differences among the field samples revealed some correlations in their geographic and temporal distributions. Specific subtypes, such as A.1, were predominantly found in the North, whereas A.4 dominated in the South of the 2400 km^2^ spanning sampling area. B and C types were found in the North and the South. Such geographic footprints could be used in future studies to better understand the dynamics and interaction of LdMNPV and its host, when another outbreak of *L. dispar* is followed by an LdMNPV epizootic, which typically occurs in the family Lymantriidae (Cory and Myers 2003). Though the sampling number in 2019 was rather low compared to 2020, there is clear evidence from one block (O) that the same subtype combinations appeared in both sampling years (Table 4, Table S5), hinting at the persistence of certain subtypes or their mixtures in the field. This finding indicates that the aNGS method is suitable for studying the population dynamics within baculovirus populations on a molecular level.

The strength of the aNGS approach as presented in this study is not only the positional identification of genome differences between different samples but also the analyses and modelling of their quantitative distribution, as well as its suitability for large sample sizes. The next *L. dispar* outbreak is expected in the investigated region and elsewhere in Bavaria from 2027 onward. The developed aNGS methodology, as well as the data established for the LdMNPV epizootics from 2019 and 2020, will provide the unique opportunity to study the long-term transmission and micro-evolutionary trends of a baculovirus infecting an insect host with wide population cycling periodicities.

## Conclusion

A robust and cost-effective strategy for analysing the genetic diversity and population structure of LdMNPV directly from *L. dispar* larvae collected in the field was developed. We identified a minimum number of SNP markers that can analyse the population structure of LdMNPV. We used high-resolution SNP frequency analysis and linear programming-based modelling to resolve complex variant mixtures, identify pure subtypes, and infer the geographic distribution and stability of viral variants over time. This approach overcomes many limitations of classical methods due to its minimal invasiveness and robust PCR and linear programming technique. It provides a valuable framework for monitoring baculovirus population dynamics and concludes the evolutionary trends of viral subtypes across space and time in local populations. Consistency with WGS validates the reliability of the amplicon-based strategy. Furthermore, we found many isolates very closely related to the biopesticide Gypchek®. As future outbreaks of *L. dispar* occur, this framework will enable in-depth tracking of LdMNPV diversity, supporting sustainable biocontrol practices and advancing our understanding of (baculo)virus–host co-evolution in natural ecosystems.

## Supplementary Material

Supplement_R1_veaf061

## Data Availability

All Illumina and Nanopore sequencing data generated in this study were deposited in NCBI Sequence Read Archive (SRA) under BioProject PRJNA1238195. The Python code and all datasets used for modelling the composition of mixed subtype samples are available on Github on https://github.com/wennj/ldmnpv-population-structure.
