# Supplementary material for: Amplicon-based analyses of single-nucleotide polymorphisms reveal the genetic structure of a forest insect baculovirus
Source: Virus Evol. 2025 Aug 23;11(1):veaf061. doi: 10.1093/ve/veaf061 (PMC12522038; doi:10.1093/ve/veaf061)
Supplement: Supplement_R1_veaf061 [file supplement_r1_veaf061.docx]

**Supplementary Material**

| Table S1: Overview of sampling of *Lymantria dispar* larvae in April/May 2019 and 2020. Forests close the given location were chosen as sampling sites. (Figure 1). | | | | | | |
| --- | --- | --- | --- | --- | --- | --- |
| Collection site | Location | Year | Month | No. of samples not sprayed* | No. of samples sprayed* | Total no. of samples |
| A | Donnersdorf, Iphofen | 2019 | April | 1 | 10 | 20 |
|  |  | 2019 | May | 9 | 0 |  |
| B | Iphofen | 2019 | April | 5 | 15 | 35 |
|  |  | 2019 | May | 15 | 0 |  |
| D | Scheinfeld | 2019 | May | 9 | 0 | 9 |
| F | Sugenheim, Ipsheim | 2019 | May | 20 | 5 | 25 |
| G | Bad Windsheim | 2019 | April | 1 | 10 | 33 |
|  |  | 2019 | May | 19 | 0 |  |
|  |  | 2020 | May | 3 | 0 |  |
| H | Uffenheim, Markt Nordheim | 2019 | April | 15 | 6 | 21 |
| J | Riedenheim, Aub | 2019 | May | 18 | 0 | 22 |
|  |  | 2020 | May | 4 | 0 |  |
| M | Wonfurt, Donnersdorf | 2019 | April | 10 | 0 | 10 |
|  |  | 2019 | May | 7 | 5 | 12 |
| N | Donnersdorf | 2019 | April | 7 | 10 | 46 |
|  |  | 2019 | May | 15 | 4 |  |
|  |  | 2020 | May | 10 | 0 |  |
| O | Volkach | 2019 | April | 0 | 10 | 20 |
|  |  | 2019 | May | 10 | 0 |  |
| S | Theres | 2019 | May | 9 | 0 | 19 |
|  |  | 2020 | May | 10 | 0 |  |
| T | Bad Windsheim | 2019 | May | 16 | 0 | 16 |
| * Treated with insecticide Mimic® | | | | | | |

| Table S2: Statistics on Illumina reads of whole genome sequencing (WGS) of the LdMNPV samples of this study. Reads mapping to reference LdMNPV-5-6 (GenBank accession no. AF081810) were used to determine single nucleotide polymorphism (SNP) positions. The raw Illumina sequencing data of the NCBI BioSamples are deposited under BioProject PRJNA1238195. | | | | | |
| --- | --- | --- | --- | --- | --- |
| LdMNPV sample | NCBI BioSample | Total paired reads sequenced | No. quality filtered paired reads | No. reads mapping to LdMNPV-5-6 | LdMNPV mean read depth SD |
| SB | SAMN47462188 | 2,829,544 | 2,564,972 | 2,383,918 | 1,950 ± 351 |
| SU | SAMN47462189 | 3,573,802 | 3,224,466 | 3,139,112 | 2,445 ± 513 |
| SZ | SAMN47462190 | 3,051,626 | 2,751,156 | 2,675,706 | 2,156 ± 480 |
| Gyp | SAMN47462191 | 2,542,118 | 2,279,120 | 2,262,625 | 1,796 ± 408 |

| Table S3. Read statistics on the Nanopore-sequenced PCR amplicons. Unfiltered and filtered for Q > 7 amount, quality and length of produced reads in the MinION sequencing | | | | | | | |
| --- | --- | --- | --- | --- | --- | --- | --- |
| LdMNPV sample | Locality | Total reads | | Mean read quality | | Mean read length | |
|  |  | unfiltered | filtered  (Q > 7) | unfiltered | filtered (Q > 7) | unfiltered | filtered (Q > 7) |
| Gyp | - | 364,486 | 337,724 (92.7% | 11.6 | 12.1 | 395 | 381 |
| SZ | Sulzheim | 440,470 | 411,431 (93.4%) | 11.7 | 12.2 | 437 | 429 |
| SU | Sugenheim | 379,701 | 349,325 (92.0% | 11.5 | 12 | 423 | 416 |
| SB | Schraudenbach | 267,945 | 250,753 (93.6%) | 11.7 | 12.1 | 387 | 386 |

| Table S4. Mean read counts and proportions of different amplicons for raw data and trimmed data (Q >30) Illumina Miseq generated reads, split by the primer pairs, that generated the fragment. | | | | |
| --- | --- | --- | --- | --- |
| Primer pair (amplicon) | raw data | | trimmed data Q>30 | |
|  | mean count [reads] | mean count [%] | mean count [reads] | mean count [%] |
| #2 | 44,337 ± 14,921 | 43.2 | 43,576 ± 14,743 | 43.6 |
| #6 | 9,121 ± 3,077 | 8.9 | 8,608 ± 2,949 | 8.6 |
| #9 | 21,099 ± 8,080 | 20.5 | 20,281 ±7,647 | 20.3 |
| #11 | 18,176 ± 9,837 | 17.7 | 17,958 ± 9,726 | 18.0 |
| #12 | 10,047 ± 6,199 | 9.8 | 9,634 ± 5,889 | 9.6 |

Table S5: Overview on four mixed samples (Gyp to SZ) and 70 single larva samples (23 to 113) of LdMNPV. Given are the clusters A to C the samples were allocated to, if they were pure (>95%) or mixed, the year of sampling, the collection block [orange=north, blue =south], and the modelled frequency of subtypes A.1 to C.1 in the different samples [pink = 0.05-0.1, orange = 0.1-0.95, green = >0.95]. The largest absolute entry of the error matrix F is shown as “maximum error” for each sample. The last column shows all positions of the 21 SNPs with errors (i.e. absolute entries of the error matrix F) >0.1 (compare Figure 7) derived from the linear model.

| **Sample** | **Cluster** | **Mixture** | **Sampling Year** | **Sampling Block** | **A.1** | **A.2** | **A.3** | **A.4** | **B.1** | **B.2** | **B.3** | **C.1** | **Maximum error** | **Positions with errors >10%** |
| --- | --- | --- | --- | --- | --- | --- | --- | --- | --- | --- | --- | --- | --- | --- |
| Gyp | A | y |  | Gyp | 0.1580 | 0 | 0.5075 | 0.1014 | 0 | 0.1849 | 0.0026 | 0.0454 | 0.2757 | 13: 0.1101, 18: 0.2688, 19: 0.1827, 21: 0.2757, |
| SB | A | Y |  | SB | 0.6219 | 0 | 0.0006 | 0 | 0.0691 | 0.2017 | 0.1046 | 0.0011 | 0.2297 | 6: 0.2027, 7: 0.1984, 9: 0.2041, 19: 0.2297, 20: 0.1708, |
| SU | B | Y |  | SU | 0.0083 | 0 | 0 | 0 | 0 | 0.4476 | 0.5413 | 0.0012 | 0.5024 | 14: 0.5024, 20: 0.1833, 21: 0.1168, |
| SZ | C | y |  | SZ | 0.1019 | 0 | 0 | 0 | 0 | 0.0022 | 0.0028 | 0.8902 | 0.8966 | 8: 0.3709, 11: 0.7133, 12: 0.6321, 13: 0.2207, 14: 0.8966, 16: 0.1961, 17: 0.7683, 18: 0.8928, 20: 0.6973, 21: 0.1898, |
| 23 | B | y | 2019 | G | 0.0760 | 0 | 0 | 0 | 0.9005 | 0.0154 | 0 | 0.0074 | 0.5545 | 14: 0.5545, |
| 24 | C | y | 2019 | H | 0.0826 | 0 | 0 | 0 | 0.0036 | 0 | 0 | 0.9128 | 0.9960 | 11: 0.9907, 12: 0.996, 14: 0.9078, |
| 26 | A | n | 2019 | O | 0.9884 | 0.0053 | 0 | 0 | 0.0013 | 0.0019 | 0 | 0.0022 | 0.0099 |  |
| 34 | A | y | 2019 | O | 0.7606 | 0 | 0.0007 | 0 | 0.1016 | 0.0076 | 0.1266 | 0.0022 | 0.1433 | 14: 0.1416, 19: 0.1433, 20: 0.1414, 21: 0.1359, |
| 35 | B | y | 2019 | O | 0.4097 | 0 | 0 | 0 | 0.0692 | 0.2329 | 0.2854 | 0.0017 | 0.3356 | 6: 0.2347, 7: 0.1925, 9: 0.2273, 14: 0.3356, 19: 0.3093, |
| 36 | A | y | 2020 | S | 0.8383 | 0 | 0.0008 | 0.0395 | 0 | 0.0009 | 0.0342 | 0.0862 | 0.1072 | 21: 0.1072, |
| 37 | A | y | 2020 | S | 0.8957 | 0.0975 | 0.0005 | 0 | 0.0004 | 0.0021 | 0 | 0.0032 | 0.1181 | 11: 0.1181, 14: 0.102, |
| 40 | A | n | 2020 | S | 0.0022 | 0.9923 | 0 | 0 | 0.0017 | 0.0017 | 0 | 0.0015 | 0.0050 |  |
| 41 | B | n | 2020 | N | 0.0011 | 0.0034 | 0 | 0 | 0.9929 | 0 | 0 | 0.0020 | 0.0345 |  |
| 42 | B | y | 2020 | N | 0.3256 | 0 | 0.0002 | 0 | 0.6664 | 0.0022 | 0 | 0.0051 | 0.2609 | 18: 0.139, 19: 0.2198, 20: 0.2609, 21: 0.2229, |
| 43 | A | y | 2020 | N | 0.1902 | 0.7442 | 0.0067 | 0 | 0.0387 | 0.0068 | 0 | 0.0124 | 0.0504 |  |
| 44 | B | n | 2020 | N | 0.0012 | 0.0018 | 0.0008 | 0 | 0.9939 | 0 | 0 | 0.0016 | 0.0289 |  |
| 45 | B | y | 2020 | N | 0.4652 | 0 | 0.0139 | 0 | 0.3859 | 0.0109 | 0.1180 | 0.0055 | 0.4395 | 1: 0.4377, 2: 0.4395, 4: 0.4395, 6: 0.4383, 9: 0.4092, 14: 0.1345, 18: 0.1934, 20: 0.1131, |
| 47 | A | y | 2020 | S | 0.5072 | 0 | 0.0126 | 0.0094 | 0.2210 | 0.0168 | 0.1778 | 0.0544 | 0.4062 | 18: 0.4062, 19: 0.359, 20: 0.3752, 21: 0.3981, |
| 48 | A | n | 2020 | S | 0.0004 | 0.9939 | 0.0009 | 0 | 0.0017 | 0.0014 | 0 | 0.0012 | 0.0031 |  |
| 51 | A | n | 2020 | J | 0 | 0 | 0.9946 | 0 | 0.0011 | 0.0019 | 0 | 0.0017 | 0.0059 |  |
| 52 | A | n | 2020 | B | 0.0084 | 0.0069 | 0.0006 | 0.9783 | 0.0004 | 0.0035 | 0 | 0.0014 | 0.0077 |  |
| 53 | A | n | 2020 | B | 0.0124 | 0.0041 | 0.0004 | 0.9737 | 0.0055 | 0 | 0 | 0.0034 | 0.0069 |  |
| 54 | A | n | 2020 | D | 0.0440 | 0.0057 | 0.0037 | 0.9170 | 0.0080 | 0.0018 | 0 | 0.0192 | 0.0165 |  |
| 55 | A | n | 2020 | D | 0.0138 | 0.0108 | 0.0011 | 0.9622 | 0.0009 | 0.0075 | 0 | 0.0025 | 0.0102 |  |
| 56 | A | n | 2020 | D | 0.0241 | 0 | 0 | 0.9534 | 0 | 0.0135 | 0.0068 | 0.0011 | 0.0152 |  |
| 57 | A | n | 2020 | D | 0.0020 | 0.0084 | 0.0006 | 0.9823 | 0.0022 | 0.0020 | 0 | 0.0020 | 0.0066 |  |
| 58 | A | n | 2020 | D | 0.0196 | 0 | 0.0020 | 0.9581 | 0.0142 | 0.0036 | 0 | 0.0019 | 0.0132 |  |
| 59 | A | n | 2020 | D | 0.0039 | 0.0122 | 0.0006 | 0.9760 | 0.0034 | 0.0013 | 0 | 0.0019 | 0.0065 |  |
| 62 | A | n | 2020 | O | 0.9296 | 0 | 0.0007 | 0 | 0 | 0.0031 | 0.0066 | 0.0595 | 0.0572 |  |
| 63 | A | y | 2020 | O | 0.8258 | 0 | 0 | 0 | 0 | 0.0713 | 0.0986 | 0.0030 | 0.1392 | 19: 0.1392, |
| 64 | A | y | 2020 | O | 0.8617 | 0 | 0.0022 | 0 | 0 | 0.0468 | 0.0868 | 0.0018 | 0.0934 |  |
| 65 | A | y | 2020 | O | 0.8805 | 0 | 0 | 0 | 0.0529 | 0.0101 | 0.0425 | 0.0103 | 0.1262 | 1: 0.1149, 2: 0.1167, 4: 0.1176, 6: 0.1262, 9: 0.1207, |
| 66 | A | n | 2020 | O | 0.9966 | 0 | 0 | 0 | 0.0010 | 0.0007 | 0 | 0.0011 | 0.0030 |  |
| 67 | A | y | 2020 | O | 0.7184 | 0 | 0.0014 | 0 | 0 | 0.1292 | 0.1279 | 0.0216 | 0.1694 | 14: 0.1543, 19: 0.1694, |
| 68 | A | y | 2020 | O | 0.6887 | 0 | 0.0006 | 0 | 0 | 0.1365 | 0.0791 | 0.0946 | 0.1690 | 3: 0.136, 5: 0.137, 6: 0.1359, 13: 0.1215, 14: 0.1564, 19: 0.169, |
| 69 | A | n | 2020 | O | 0.9925 | 0 | 0.0006 | 0 | 0.0021 | 0.0025 | 0 | 0.0015 | 0.0057 |  |
| 70 | A | n | 2020 | O | 0.9858 | 0 | 0.0008 | 0 | 0.0011 | 0.0104 | 0 | 0.0012 | 0.0104 |  |
| 71 | A | n | 2020 | O | 0.9592 | 0.0064 | 0 | 0.0233 | 0 | 0.0010 | 0.0018 | 0.0077 | 0.0301 |  |
| 72 | A | y | 2020 | O | 0.4425 | 0.3024 | 0 | 0.0842 | 0.0800 | 0.0408 | 0 | 0.0495 | 0.1295 | 18: 0.1272, 19: 0.1181, 21: 0.1295, |
| 76 | A | n | 2020 | N | 0.9947 | 0 | 0.0004 | 0 | 0.0013 | 0.0011 | 0 | 0.0019 | 0.0049 |  |
| 77 | C | y | 2020 | N | 0.0341 | 0.0089 | 0.0021 | 0 | 0.0255 | 0.0488 | 0.0142 | 0.8654 | 0.1483 | 8: 0.1483, |
| 78 | B | y | 2020 | N | 0.0948 | 0 | 0.0476 | 0 | 0.2058 | 0.1008 | 0.2928 | 0.2572 | 0.3733 | 8: 0.254, 9: 0.2296, 14: 0.3733, 16: 0.1742, 18: 0.1455, 19: 0.1598, |
| 79 | A | y | 2020 | N | 0.4196 | 0 | 0.0747 | 0 | 0.0668 | 0.1051 | 0.1434 | 0.1874 | 0.1847 | 8: 0.1847, 14: 0.1528, 19: 0.1361, |
| 80 | A | n | 2020 | N | 0.9855 | 0.0087 | 0 | 0 | 0.0010 | 0.0020 | 0 | 0.0021 | 0.0139 |  |
| 81 | B | y | 2020 | S | 0.0860 | 0 | 0.0042 | 0.0129 | 0.0495 | 0.8025 | 0.0277 | 0.0153 | 0.0764 |  |
| 83 | A | y | 2020 | S | 0.0501 | 0 | 0.7868 | 0.0106 | 0.0738 | 0.0117 | 0.0380 | 0.0277 | 0.0269 |  |
| 84 | A | y | 2020 | S | 0.5607 | 0.0308 | 0.0348 | 0 | 0.1488 | 0.0273 | 0.0352 | 0.1597 | 0.1066 | 8: 0.1066, |
| 85 | A | y | 2020 | S | 0.9132 | 0 | 0.0093 | 0 | 0.0083 | 0.0146 | 0 | 0.0529 | 0.0499 |  |
| 86 | A | y | 2020 | J | 0.4272 | 0 | 0.0335 | 0 | 0.1594 | 0.0958 | 0.0779 | 0.2023 | 0.1036 | 18: 0.1036, |
| 87 | A | y | 2020 | J | 0.8416 | 0 | 0 | 0 | 0.0145 | 0.0076 | 0.0700 | 0.0652 | 0.1339 | 18: 0.1339, |
| 90 | A | y | 2020 | N | 0.6131 | 0.0382 | 0 | 0.2555 | 0.0026 | 0.0832 | 0 | 0.0066 | 0.0506 |  |
| 91 | C | y | 2020 | N | 0.0573 | 0 | 0.0021 | 0 | 0.0057 | 0.3277 | 0.0201 | 0.5848 | 0.5373 | 8: 0.5373, 16: 0.2202, 18: 0.3094, 19: 0.1882, 21: 0.2921, |
| 92 | C | y | 2020 | N | 0.0901 | 0 | 0 | 0 | 0 | 0.1438 | 0.0121 | 0.7532 | 0.4704 | 8: 0.4704, 14: 0.1786, 16: 0.1014, |
| 93 | C | n | 2020 | N | 0.0012 | 0.0022 | 0.0008 | 0 | 0.0026 | 0.0010 | 0.0001 | 0.9918 | 0.0107 |  |
| 94 | A | y | 2020 | N | 0.8870 | 0 | 0.0033 | 0 | 0 | 0.0596 | 0.0218 | 0.0279 | 0.0656 |  |
| 95 | C | n | 2020 | N | 0.0030 | 0.0007 | 0 | 0.0016 | 0.0018 | 0 | 0 | 0.9921 | 0.0226 |  |
| 96 | A | n | 2020 | N | 0.0007 | 0.0111 | 0.0003 | - | 0.0012 | 0.0009 | 0 | 0.0021 | 0.0081 |  |
| 97 | A | n | 2020 | N | 0.0016 | 0.009 | 0 | - | 0.0013 | 0.0009 | 0 | 0.0040 | 0.0060 |  |
| 98 | C | n | 2020 | N | 0.0001 | 0.0021 | 0.0011 | - | 0.0020 | 0 | 0 | 0.9926 | 0.0196 |  |
| 99 | C | y | 2020 | F | 0.0147 | 0 | 0 | 0.3603 | 0 | 0.1130 | 0.0019 | 0.5097 | 0.4770 | 3: 0.1115, 5: 0.1103, 6: 0.1096, 8: 0.477, 13: 0.1407, 19: 0.1107, |
| 100 | A | y | 2020 | F | 0.0175 | 0.0175 | 0 | 0.5565 | 0.0055 | 0.0023 | 0 | 0.4000 | 0.3866 | 8: 0.3866, 21: 0.1533, |
| 101 | C | y | 2020 | F | 0.0310 | 0 | 0.0006 | 0.4100 | 0 | 0.0020 | 0.0024 | 0.5530 | 0.5279 | 8: 0.5279, 13: 0.1796, 16: 0.1536, 21: 0.1189, |
| 102 | C | y | 2020 | F | 0.0139 | 0 | 0 | 0.2417 | 0 | 0.1006 | 0.0004 | 0.6426 | 0.5968 | 3: 0.1013, 8: 0.5968, 13: 0.1155, |
| 103 | C | y | 2020 | F | 0.0112 | 0 | 0 | 0.3719 | 0 | 0.0596 | 0.0011 | 0.5555 | 0.5158 | 8: 0.5158, |
| 104 | B | n |  | SB | 0.0007 | 0.0051 | 0.0007 | 0 | 0.0018 | 0.9899 | 0 | 0.0016 | 0.0061 |  |
| 105 | C | n |  | SB | 0.0051 | 0.0001 | 0.0010 | 0.0004 | 0.0025 | 0 | 0 | 0.9907 | 0.0363 |  |
| 106 | B | n |  | SU | 0.0027 | 0.0013 | 0 | 0.0009 | 0.9930 | 0 | 0 | 0.0014 | 0.0365 |  |
| 107 | B | n |  | SU | 0.0096 | 0 | 0.0009 | 0 | 0 | 0.0252 | 0.962 | 0.0020 | 0.0619 |  |
| 108 | B | y |  | SU | 0 | 0 | 0.0517 | 0 | 0.0539 | 0.8724 | 0.019 | 0.0020 | 0.9239 | 10: 0.8737, 14: 0.9239, 21: 0.9191, |
| 109 | C | y |  | SZ | 0.0192 | 0.0175 | 0.0007 | 0.0015 | 0 | 0.5842 | 0.005 | 0.3714 | 0.6014 | 8: 0.1711, 16: 0.1039, 18: 0.6014, 21: 0.5943, |
| 110 | A | n |  | SZ | 0.0031 | 0.0165 | 0.9409 | 0 | 0.0248 | 0.0025 | 0.007 | 0.0044 | 0.0111 |  |
| 111 | A | n |  | SZ | 0.9310 | 0 | 0.0007 | 0 | 0 | 0.0184 | 0.001 | 0.0478 | 0.0653 |  |
| 112 | A | y |  | SZ | 0.4964 | 0 | 0.0006 | 0 | 0 | 0.2956 | 0.206 | 0.0011 | 0.4900 | 6: 0.2948, 7: 0.2933, 9: 0.2892, 14: 0.1831, 18: 0.1943, 19: 0.49, 20: 0.2045, 21: 0.1956, |
| 113 | A | y |  | SZ | 0.0071 | 0 | 0 | 0 | 0 | 0.4444 | 0.546 | 0.0017 | 0.5021 | 6: 0.1818, 7: 0.1777, 9: 0.178, 14: 0.5021, 18: 0.3726, 20: 0.4144, 21: 0.4099, |


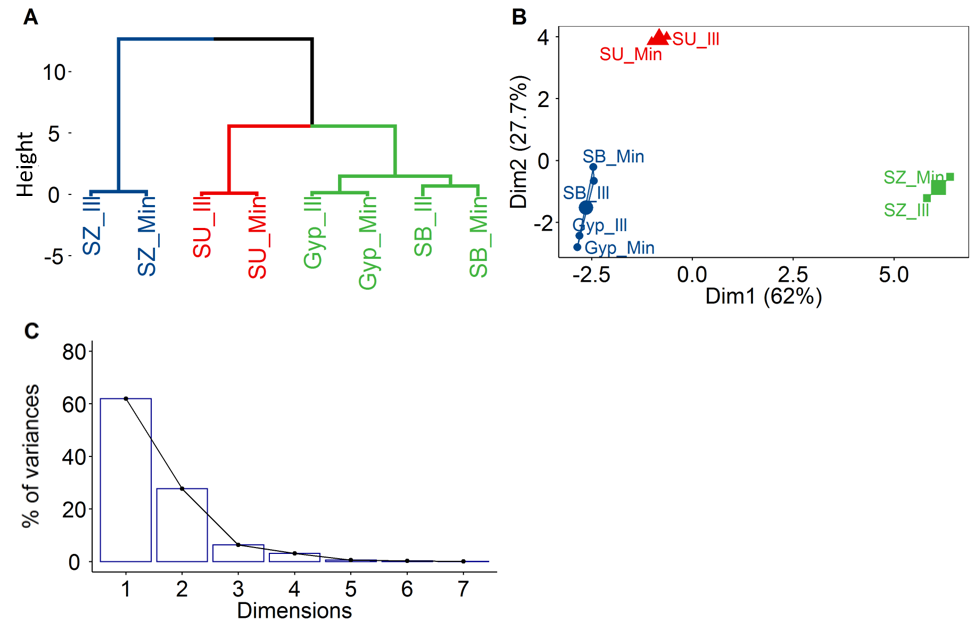


Figure S1: Hierarchical clustering on principal components of the five amplicon data set (21 SNPs) sequenced with Illumina (_Ill) and the MinION device (_Min) of SB, SZ, SU and Gypchek (Gyp). A) Hierarchical clustering on principal component. With Ward`s method three clusters could be determined. B) Classification of the genome data into three clusters was supported by a k-means consolidation. C) The bar graph shows the percentage of variances explained by each dimension. Here, the MinION and Illumina data was analysed together to test if the PCR itself or the use of Nanopore sequenceing vs. Illumina sequencing would introduce a structural bias into the analysis. The hypothesis was if the MinION sequenced data and the Illumina sequenced data of the respective samples are showing the least similarity in the factor map, the bias introduced is not essential. The same 58 SNP positions were analysed between Illumina and MinION data. For each of the combination of primer pairs a HCPC was performed. If the pattern of similarity is identical to the reference pattern (Figure 3), the specific combinations of primer pairs was considered as a positive hit.


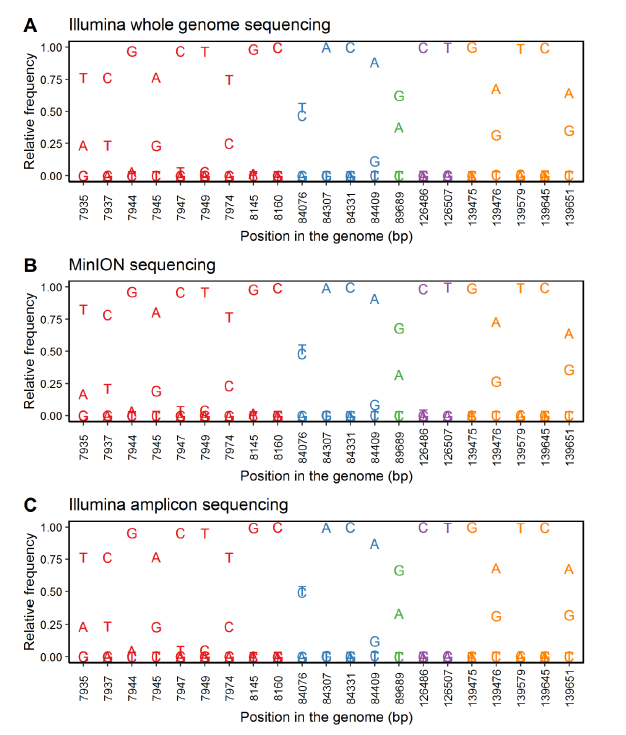


**Figure S2**: Comparison of SNP frequency plots of LdMNPV-Gyp generated from **A**) Illumina whole genome sequencing, **B**) Nanopore Amplicon Next Generation Sequencing (aNGS), and **C**) Illumina aNGS. Note the highly similar SNP frequency distribution indicating the robustness of the aNGS.
